# Emergence of autochthonous *Leishmania infantum* infection in dogs from Costa Rica confirmed by multimodal diagnostics: a case series

**DOI:** 10.3389/fvets.2025.1704403

**Published:** 2026-01-21

**Authors:** Víctor M. Montenegro, Leticia Cajal-Omella, Josué Campos-Camacho, Javier Jiménez-Tuk, Carlos Mata-Somarribas, Alejandro Alfaro-Alarcón, Mariana Guevara-González, Paula Peña, Joban Quesada, Luis M. Romero-Vega, Alicia Rojas

**Affiliations:** 1 Laboratory of Parasitology, School of Veterinary Medicine, Universidad Nacional de Costa Rica, Heredia, Costa Rica; 2 Veterinaria Cavallini, Guanacaste, Costa Rica; 3 Laboratorio de Patologia Veterinaria LAPAVET-ESFA, Catedra de PatologÍa e HistologÍa, Escuela de Medicina y CirugÍa Veterinaria San Francisco de AsÍs, San José, Costa Rica; 4 Clínica Veterinaria Dr. Javier Jiménez Tuk, Heredia, Costa Rica; 5 Laboratório de Pesquisa em Leishmanioses (LPL), Programa de Pós-graduação em Biologia Celular e Molecular, Instituto Oswaldo Cruz, Rio de Janeiro, Brazil; 6 Department of Pathology, School of Veterinary Medicine, Universidad Nacional, Heredia, Costa Rica; 7 Berlin Institute of Health, Institute of Virology, Charité-Universitätsmedizin Berlin, Corporate Member of Freie Universität Berlin, Humboldt-Universität zu Berlin, Berlin, Germany; 8 Laboratory of Helminthology, Faculty of Microbiology, University of Costa Rica, San José, Costa Rica; 9 Institute of Pathology, University of Veterinary Medicine Hannover Foundation, Hannover, Germany; 10 Centro de Investigación en Enfermedades Tropicales, University of Costa Rica, San José, Costa Rica

**Keywords:** protozoa, canine visceral leishmaniasis, zoonosis, One Health, case report

## Abstract

**Background:**

Canine visceral leishmaniasis (CVL) is a vector-borne zoonotic disease caused by *Leishmania infantum*. This parasite has been reported in humans and dogs from Costa Rica over the past four decades as sporadic reports. In this study, we analyzed eight cases of autochthonous infections in dogs presumably originating from Santa Cruz, Guanacaste, and Santa Ana, San José, Costa Rica, none of which had a history of travel abroad.

**Methods:**

Eight dogs with suspected CVL were analyzed using serological assays (Speed Leish K® (VIRBAC Diagnostics, France) or Antigen Rapid CaniV-4 (Leish)® (BIONOTE, Mexico)), five dogs were detected in 2023, and three during 2025. Histopathological staining was applied in cases with spleen, dermal, and lymph node involvement to determine the presence of *Leishmania* amastigotes. Blood, lymph node aspirates, conjunctival swabs, or cutaneous lesion swabs were also analyzed for the presence of *Leishmania* spp. ITS1, hsp70, and kDNA fragments. Phylogenetic and haplotype network analyses were conducted for hsp70 and kDNA data.

**Results:**

Four dogs showed various clinical manifestations that included persistent anemia, thrombocytopenia, splenomegaly, exfoliative dermatitis, and onychogryphosis, whereas the other four dogs remained subclinical or asymptomatic. Histopathological analysis revealed numerous intracellular amastigotes in lymph node aspirates, spleen sections, and ear skin biopsy. Moreover, seven out of eight dogs were positive in the serological analysis, and the other seven to the *Leishmania* ITS1 PCR. Phylogenetic analysis of kDNA fragments revealed that sequences derived from our country clustered with those of *L. infantum* from the Old World, rather than with ones from Brazil, indicating a likely introduction from outside the Americas. All infected dogs received allopurinol and, when available, also meglumine antimoniate.

**Conclusion:**

Infection with *L. infantum* in Costa Rican dogs was confirmed by clinical and laboratory evidence and thus represents the first autochthonous cases of CVL in our country. This study highlights the urgent need for routine canine testing, sandfly surveillance, access to proper treatments, and increased awareness, emphasizing the importance of public health policies for controlling leishmaniasis in both animals and humans from a One Health perspective.

## 1 Introduction

*Leishmania* is a genus comprising over 50 species, with approximately 20 pathogenic to vertebrates (1, 2). Each year, up to a million people in tropical and subtropical regions are affected, with two-thirds of the burden occurring in the Americas (3–5). Clinical manifestations in humans vary according to species; *Leishmania infantum* is among the most important zoonotic species and the primary cause of visceral leishmaniasis in the Americas (6). Dogs serve as reservoirs of *L. infantum,* often carrying subclinical infections that sustain parasite transmission, without evident pathology, complicating diagnosis and control (7). Differences between New and Old World *L. infantum* strains have been reported, including variations in host preference and pathology. While *L. infantum* is classically linked to visceral disease, Old World strains can also produce cutaneous lesions (7, 8). In the New World, atypical cutaneous leishmaniasis associated with *L. infantum* has been documented in Venezuela (9), Brazil (10) Honduras (11), and Costa Rica (12), highlighting its broader clinical spectrum and epidemiological importance.

In Costa Rica, *Leishmania panamensis* and *Leishmania braziliensis* have been reported as causative agents of cutaneous leishmaniasis in humans (13, 14). On the other side, *L. infantum* has been documented only four times. The first case involved a domestic dog imported from Spain (15). The second and third reports were made on humans and included an outbreak of atypical cutaneous leishmaniasis in Liberia, Guanacaste, in 1989 (12) and a visceral leishmaniasis case in an immunocompetent child in 1999 (16). Moreover, the fourth report involved captive snakes in a serpentarium (17). To date, no confirmed cases of autochthonous canine visceral leishmaniasis (CVL) have been documented in Costa Rica, despite sporadic reports of *L. infantum* in other hosts. Therefore, this study aimed to confirm the presence of autochthonous infections in domestic dogs from Guanacaste using clinical, serological, histopathological, and molecular approaches. This analysis underscores the urgent need for enhanced surveillance of dogs and monitoring for possible human cases by public health authorities.

## 2 Methods

### 2.1 Clinical case description

Complete demographical, clinical, laboratory and treatment information of all cases can be found in Supplementary Data Sheet 1.

#### 2.1.1 Case 1

**Figure 1:**
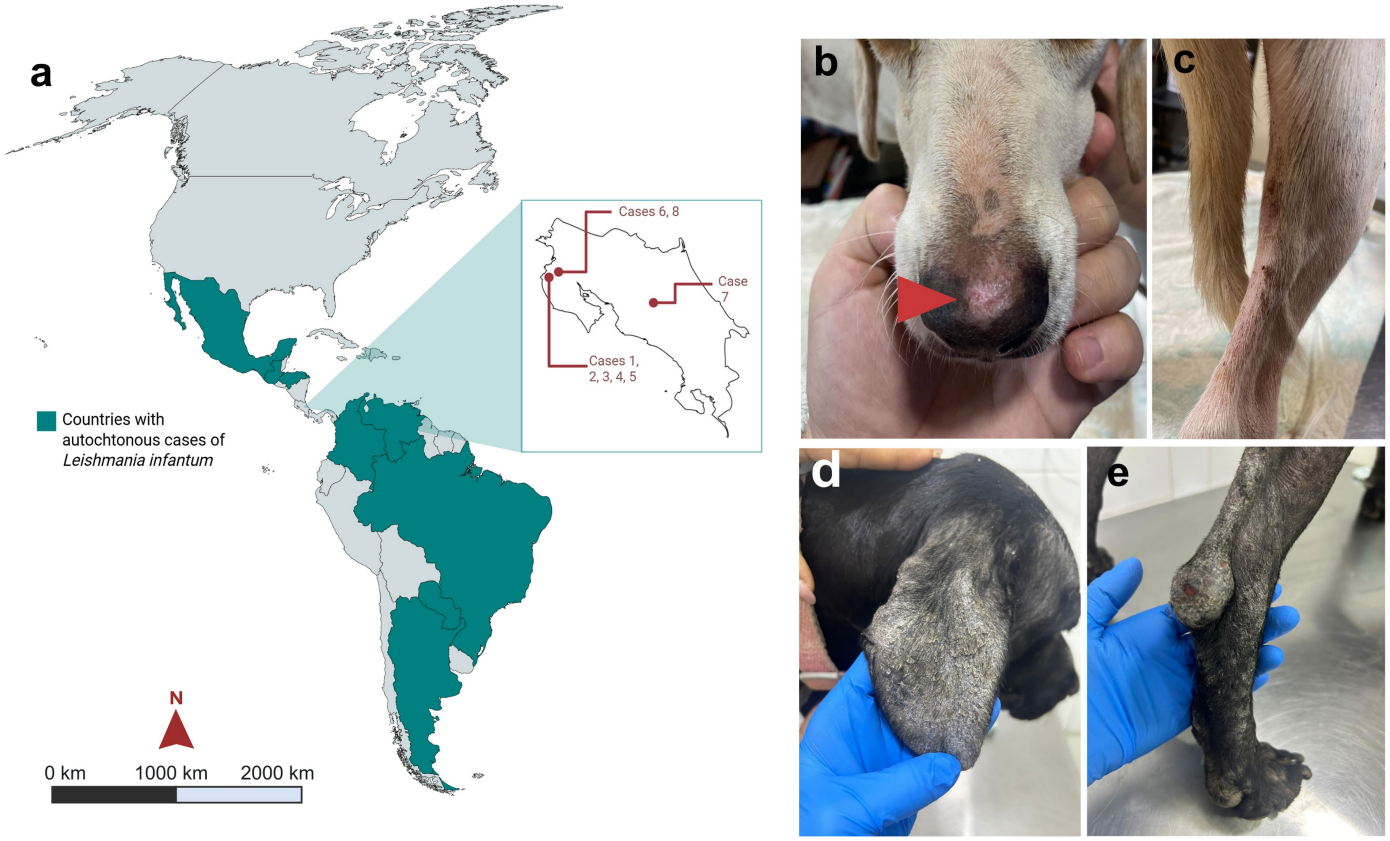
Demographic and clinical characteristics of cases analyzed in this study. **(a)** Map of the Americas shows those geographical regions with reported autochthonous cases according to Burza et al. (1). Clinical presentation of cases 1 and 6 with visceral leishmaniasis. **(b)** Clinical examination of case 1 from Guanacaste, Costa Rica, showing an ulcer in the dog’s snout (red triangle) and **(c)** hind leg alopecia. Samples were drawn from both anatomical locations and lesions. Case 6 presented scaling, alopecia, hyperkeratosis, and crusts on the skin of the ears **(d)**, snout, and hind legs **(e)**.

A 4-year-old, 14 kg mongrel dog from Santa Cruz, Guanacaste (10°17′20”N, 85°49′10”W) (Figure 1a) was presented to a veterinary hospital in September 2023 with weight loss, cachexia, a snout ulcer, and hind leg alopecia (Figures 1b,c). Hematological analysis revealed severe anemia, thrombocytopenia, and marked azotemia. Serum was tested for *L. infantum* antibodies, and a lymph node aspirate was obtained from the right popliteal node for cytology. Additional samples included EDTA blood, lymph node aspirates, a skin biopsy from the hind leg, and swabs from the nasal lesions and conjunctiva, which were all analyzed for *Leishmania* DNA. Treatment consisted of subcutaneous meglumine antimoniate (Glucantime, Merial, Germany) for 28 days at 100 mg/kg, plus oral allopurinol (Laboratorios Normon, Spain) for 2 months at 10 mg/kg twice daily. During follow-up in November 2023, the dog was lost in its household during follow-up, preventing further clinical evaluation.

#### 2.1.2 Cases 2, 3, 4, and 5

In December 2023, blood samples were collected from 23 dogs living near the household of case 1 (Figure 1a, Supplementary Data Sheet 2). Since most of the dogs showed no signs related to *Leishmania* infection, the samples were first screened using a serological test, followed by PCR testing. Of these animals, four tested positive for the serological assay. Case 2 was a 4-year-old female Schnauzer, case 3 was a 2-year-old female mongrel, case 4 was an 8-year-old male mongrel, and case 5 was a 3-year-old male mongrel. Cases 2, 3, and 4 did not show associated clinical manifestations and were identified as asymptomatic, with no further clinical history, but case 5 had a chronic skin lesion in his hind leg, which did not resolve. Of these, case 4 received treatment with allopurinol for 6 months at a dose of 10 mg/kg twice daily and subcutaneous meglumine antimoniate for 28 days at a dose of 100 mg/kg, whereas case 5 received only allopurinol at the same dose. On the other hand, owners of dogs 2 and 3 refrained from treating the dogs. As a follow-up, cases 2, 3, and 5 died from unrelated conditions.

#### 2.1.3 Case 6

In February 2025, a 5-year-old female mongrel dog was brought to the veterinary hospital due to persistent exfoliative dermatitis, alopecia, hyperkeratosis, onychogryphosis, and crust formation on the snout, ears (Figure 1d), and hind legs (Figure 1e). The dog originated from a community approximately 10 km from the locations of cases 1 to 5 in Guanacaste (10°21′4.104”N, −85°47′22.704”W; Figure 1a). A complete blood count revealed severe anemia, leukopenia, and thrombocytopenia. A serum sample was collected for serological analysis, and blood was drawn for DNA testing. Additionally, a skin scraping from the lesions was obtained for DNA extraction. The dog was treated with allopurinol for 4 months at a dose of 10 mg/kg twice daily.

#### 2.1.4 Case 7

A 4-year-old female mongrel, born in Santa Cruz, Guanacaste (10°19′59.952”N, 85°49′57.324”W), lived there during her first year before relocation to San José, Costa Rica (Figure 1a). In December 2024, she presented with exfoliative dermatitis on her ears and legs. By March 2025, lesions persisted, prompting cytological analysis of ear skin, which revealed numerous intracellular inclusions suggestive of *Leishmania* spp. Histopathology and blood PCR confirmed infection. Treatment with allopurinol (10 mg/kg, twice daily) was initiated. Follow-up indicated good body condition and resolution of skin lesions, demonstrating therapeutic efficacy.

#### 2.1.5 Case 8

In March 2025, a 4-year-old female Boxer from the same community as case 6 was presented to a clinic in San José, Costa Rica (Figure 1a). The dog exhibited marked splenomegaly, anemia, and thrombocytopenia. The spleen was completely resected and analyzed, and a blood sample was collected for DNA testing. The dog was treated with allopurinol at a dose of 10 mg/kg twice daily. Unfortunately, it died 3 days after the splenectomy.

### 2.2 Serological analysis

All samples were collected with the consent of dog owners, following veterinary ethical standards and Costa Rican animal welfare regulations. Serum samples were tested qualitatively for circulating antibodies against *L. infantum* by using the Antigen Rapid CaniV-4 (Leish)® (BIONOTE, Mexico) or the SpeedLeishK® (VIRBAC Diagnostics, France), depending on the test’s availability (Table 1). The used antigen was not specified by the manufacturer for the first kit, whereas the second assay detected *Leishmania*-specific anti-kinesin antibodies. Cases 1 and 6 were tested with the Antigen Rapid CaniV-4 (Leish)®, whereas cases 1, 2, 3, 4, and 5 were tested with the Speed Leish K® in accordance with the manufacturer’s instructions.

**Table 1:** Summary of serological and molecular analyses run on domestic dogs’ positive to *Leishmania infantum.*

Case number	Breed, Sex, Age	Clinical Signs	Geographical collection site	Serological analysis results*	ITS1 real-time PCR results (% of identity, % of coverage, closest similarity) (Accession number of the sequence derived in this study)	hsp70 PCR results (% of identity, % of coverage, closest similarity) (Accession number of the sequence derived in this study)	kDNA PCR results (% of identity, % of coverage, closest similarity) (Accession number of the sequence derived in this study)
1	Mongrel, M, ≥4 y	Weight loss, cachexia, snout ulcer, hind leg alopecia	Tamarindo, Santa Cruz, Guanacaste	Positive ARCL and SLK	*L. infantum* (100,100, MW288102) in all collected biological samples, (PQ581103, PQ581104, PQ581105, PQ581106)	Negative in all collected biological samples	Negative in all collected biological samples
2	Schnauzer, F, 4 y	None	Tamarindo, Santa Cruz, Guanacaste	Positive to SLK	*L. infantum* (100,100, MW288102) (PQ581107)	*L. infantum* (100, 100, OR136937.1)	*L. infantum* (100,99, MH605317.1) (PQ576746)**
3	Mongrel, F, 2 y	None	Tamarindo, Santa Cruz, Guanacaste	Positive to SLK	*L. infantum* (100,100, MW288102) (PQ581108)	*L. infantum* (100, 100, PP397159.1) (PQ576749)**	*L. infantum* (100,99, MH605317.1) (PQ576747)**
4	Mongrel, M, 8 y	None	Tamarindo, Santa Cruz, Guanacaste	Negative	Weak positive	*L. infantum* (99.48, 100, PP505437.1)***	*L. infantum* (100,99, MH605317.1) (PQ576748)**
5	Mongrel, M, 3 y	Chronic hind leg skin lesion	Tamarindo, Santa Cruz, Guanacaste	Positive to SLK	Not available	Not available	Not available
6	Mongrel, F, 5 y	Exfoliative dermatitis, alopecia, crusts, onychogryphosis	Tamarindo, Santa Cruz, Guanacaste	Positive to ARCL	*L. infantum* (100,100, MW288102) (PV780488)	Negative	Negative
7	Mongrel, F, 4 y	Ear and leg dermatitis	Santa Ana, San José	Not available	*L. infantum* (100,100, MW288102) (PV780487)	Weak positive	Negative
8	Boxer, F, 4 y	Splenomegaly, anemia	Tamarindo, Santa Cruz, Guanacaste	Positive to ARCL	*L. infantum* (100,100, MW288102) (PV870489)	*L. infantum* (100,99, MZ422546) PV797869	*L. infantum* (100,100, MH605317) PV785186

*Serological analyses were run using two different kits according to their availability in the country.

**This sequence was used for phylogenetic analyses.

***This sequence was not deposited in GenBank® since only a short fragment could be sequenced.

F, female; M, male; SLK, Speed Leish K® (VIRBAC Diagnostics, France); ARCL, Antigen Rapid CaniV-4 (Leish)® (BIONOTE, Mexico).

### 2.3 Histopathological and cytological analysis

Biopsies from ear skin (case 7) and spleen (case 8) were fixed with 10% buffered formalin for 48 h, and stained with hematoxylin and eosin, as well as Giemsa. In addition, lymph node aspirates were stained using the May–Grünwald–Giemsa method for histological evaluation (18). Samples were observed under a light microscope at 400x and 1,000x magnifications.

### 2.4 DNA analysis

DNA from blood and tissue samples was isolated using the DNeasy Blood and Tissue DNA extraction kit (Qiagen®, Germany). Internal transcribed spacer 1 (ITS1), kDNA, and heat shock protein 70 (hsp70) fragments were amplified to detect *Leishmania* DNA with the primers and conditions specified in Supplementary Data Sheet 3 (19–21). Amplicons were purified with Exo-SAP and Sanger sequenced (Macrogen Inc., South Korea).

Sequence identity of the six ITS1, four hsp70, and four kDNA fragments was verified with BLAST (22). Then, the generated sequences were aligned with *L. infantum* references from other geographical regions, including available Central and South American data, using the MUSCLE algorithm (23) in MEGA7 (24), and Bayesian Inference phylogenetic trees were constructed for kDNA and hsp70 data using BEAST 2.5 (25). The Tamura-Nei substitution model, identified with JModelTest 2 (26), was applied to both loci. These genes were selected because they showed higher variability, whereas the ITS1 sequences were identical between them. Alignments were uploaded to BEAUTi, and trees were inferred with 10^8^ Monte Carlo Markov chains, 10^3^ samples, and a burn-in of 10%. Next, sample convergence was verified in Tracer, trees were compiled using TreeAnnotator, and visualized in FigTree. kDNA loci revealed intraspecies variability within *L. infantum.* Therefore, data were used to construct a Templeton Crandall-Sing haplotype network with a 95% connection limit in PopArt (27), and a Nei’s genetic distance principal component analysis was constructed with GenAIEx (28). Predicted haplotypes were stratified according to country and mapped in PopArt.

## 3 Results

Serological analysis was performed in 6 of 8 cases using each test available. Moreover, 17.4% (4 of 26, i.e., cases 1, 2, 3, and 5; Wald 95% confidence interval: 1.8–32.9%) of dogs in the initial infection focus tested positive for *L. infantum* with a combination of serological and molecular data (Table 1).

**Figure 2:**
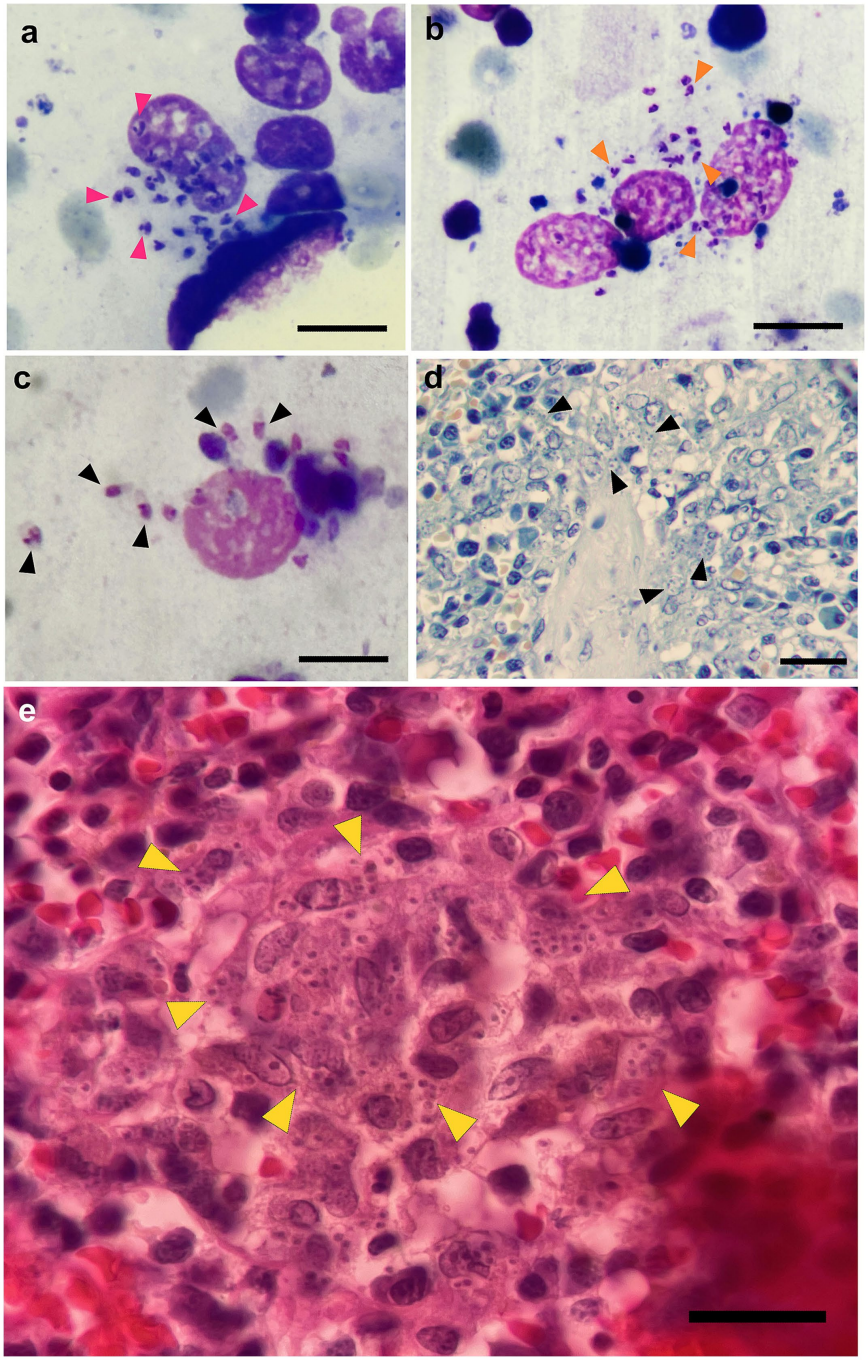
Histopathological analysis of lymph node aspirate and spleen of cases 1 and 8. **(a–c)** Amastigotes (pink, orange, and black triangles, respectively) inside monocytes obtained from case 1 popliteal lymph node aspirates (Scale bar equal 15 μm in pictures **a–c**). **(d)** Giemsa and **(e)** hematoxylin–eosin staining on spleen samples of case 6 show multiple intracellular amastigotes (black and yellow triangles, respectively). Scale bar equals 40 μm in B and 45 μm in **c**. Video of case 6 is available in Supplementary File 3.

Lymph node aspirate from case 1 showed numerous *Leishmania* spp. amastigotes within monocytes (Figure 2a). Histopathological and histochemical analysis of the ear skin biopsy from case 7 demonstrated severe granulomatous dermatitis and intracellular structures compatible with *Leishmania* sp. Analysis of case 8’s spleen revealed severe multifocal granulomatous splenitis, hyperplasia of both the white pulp and the splenic cords, and intracellular structures compatible with *Leishmania* sp. (Figures 2b–e; Supplementary Video 1).

DNA analyses of all samples from case 1 were positive for the ITS1 fragment of *Leishmania* spp., showing 100% identity with *L. infantum* (GenBank MW288102) from a sequence in Tunisia, but negative for hsp70 and kDNA PCRs (Table 1). Cases 2–5 were also ITS1-positive, with identical sequences. Blood samples from cases 2, 3, 4, and 8 were positive for hsp70, matching sequences PP397159 (Spain), PP505437, and OR136937 (Italy). kDNA fragments were obtained from cases 2, 3, 4, and 8, identical to MH605317 from a dog in Bosnia and Herzegovina. Notably, case 4 tested negative serologically, was weakly positive in ITS1 PCR, but positive for both kDNA and hsp70, highlighting discrepancies among diagnostic methods.

**Figure 3:**
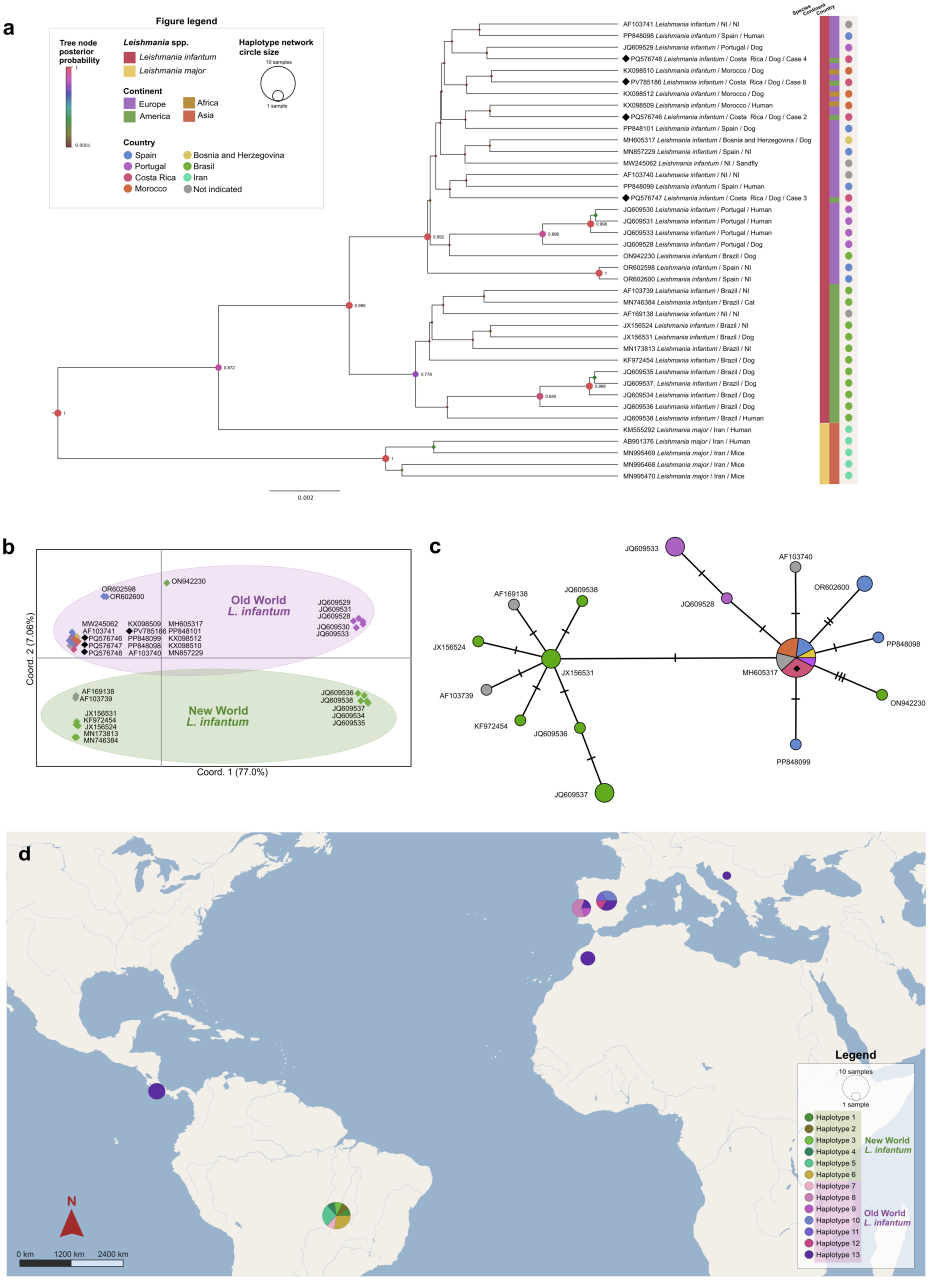
Phylogenetic analysis of *Leishmania infantum* kDNA minicircle fragment. **(a)** Bayesian inference phylogenetic tree. Node colors and sizes are proportional to the posterior probability values (PP). Sequences derived from this study are marked with a black diamond. PP values lower than 0.6 are not shown in the tree. **(b)** Principal Coordinates Analysis of Nei’s genetic distance between *L. infantum* sequences. Sequences derived from this study are written in bold letters. **(c)** Templeton Crandall from the haplotype network of *Leishmania infantum* analyzed in this study. Circle size is proportional to the number of sequences sharing the same haplotype, black nodes denote hypothetical haplotypes separating two sequences, and hatch marks correspond to the number of mutations differentiating two haplotypes. **(d)** Thirteen *Leishmania infantum* haplotypes are distributed according to geographical location.

DNA analyses revealed that all samples were *L. infantum* according to the three amplified loci. hsp70 phylogenetic tree showed sequences PQ576749 and PV797869 from Costa Rica clustering closely to sequences from Italy and Spain (Supplementary Image 1). Analysis of kDNA showed the same subdivision according to location. Sequences from Costa Rica PQ576746, PQ576747, PQ576748, and PV785186 were placed with *L. infantum* from the Old World, such as Spain, Portugal, Morocco, and Bosnia and Herzegovina, and apart from *L. infantum* from Brazil (Figure 3a), except for one sequence where no further information was provided (ON942230). Nei’s genetic distance PCA (Figure 3b) and TCS haplotype network (Figure 3c) confirmed that our sequences were placed with *L. infantum* from the Old World rather than with those from Brazil. Accordingly, all kDNA sequences derived from Costa Rica belonged to haplotype 13, together with sequences from Morocco, Spain, Portugal, and Bosnia and Herzegovina. Sequences from Brazil were grouped into haplotypes 1 to 7, which were exclusive to this country (Figure 3d).

## 4 Discussion

This study confirms active transmission of *L. infantum* in Costa Rican dogs, likely introduced through a migratory event. Even though a discrete number of sequences from Costa Rica could be obtained, phylogenetic analyses showed our isolates were closer to European and African sequences than to Brazilian ones. Although *L. infantum* has been reported from Central America in recent decades (29, 30), our findings suggest a different origin. While New World strains circulate in the region, it remains unclear whether Costa Rican cases involve newly imported parasites or previously established lineages. Previous *L. infantum* cases in dogs or humans were not subjected to molecular testing (12, 15, 16), and the possible source of infection in snakes could not be determined (17). To clarify the introduction timing and evolutionary relationships, additional Costa Rican samples and a time-calibrated phylogenetic tree using *Leishmania* fossils would provide valuable insight into the history and spread of these parasites in Central America.

Three subclinical or asymptomatic infections were identified in this study, which may contribute to parasite persistence (31). The prevalence of *L. infantum* in Costa Rica is currently unknown, but a study from 2012 sampled 146 domestic dogs from different regions of the country (32). In this analysis, samples were tested for *L. infantum* ITS1 loci, without finding any positive ones. Since then, no other screening in dogs directed to this parasite has been conducted. In the reduced survey performed in the community of the first five cases, a frequency of 17.4% was found, but it must be highlighted that a wider screening should be carried out to determine the prevalence of *L. infantum* (33). Dogs from endemic regions usually show a high risk of *L. infantum* infections, showing a large proportion of asymptomatic cases. For these reasons, domestic dogs are considered the primary peridomestic reservoirs of *L. infantum* (34). Furthermore, reports of this parasite in rodents (35), lagomorphs (36), wild canids (37), cats (38), and horses (39) can also be found, but their ecological role in the life cycle of *L. infantum* is currently unknown (34). Interestingly, the DNA of *L. infantum* was detected in snakes kept in captivity in Costa Rica without associated manifestations in 2025 (17), suggesting the role of herpetofauna as alternative reservoirs of *L. infantum*.

The clinical manifestations and histopathological findings observed in cases 1, 6, 7, and 8 were consistent with those reported for CVL (31). Some dogs infected with *L. infantum* develop clinical signs such as lymph node enlargement, skin and ear lesions, alopecia, keratoconjunctivitis, and onychogryphosis (40). Hematological tests often reveal anemia and thrombocytopenia (41), with anemia severity correlating positively with clinical signs (42). These alterations are linked to parasite density in the bone marrow, which disrupts erythropoiesis and leukopoiesis (43). Skin changes are associated with parasite replication in endothelial cells, alterations in blood vessels, and increased inflammatory infiltration, reflecting the immune system’s limited control of parasitic load. Together, these processes contribute to dermal vascularization, granulomatous inflammation, and parasite dissemination through the skin (44).

Cases in this report were confirmed using diverse diagnostic approaches, including serological assays, histopathological evaluation, and molecular methods, and inherent limitations were noticed. Although lymph node aspirates and histopathology have the highest sensitivity for detecting *L. infantum* in dogs, these samples could not be obtained from all animals due to a lack of information from clinicians. Furthermore, fatal outcomes or loss of follow-up in some cases prevented the assessment of treatment efficacy and resolution of clinical signs. Then, commercial kits from different providers were used, which, although not ideal, were necessary since kits for detecting *L. infantum* antibodies are not readily available in Costa Rica, given the absence of autochthonous or imported cases for more than two decades. One serological method lacked disclosed sensitivity and specificity; however, it identified two symptomatic dogs later confirmed by PCR, suggesting adequate performance (33). In contrast, one dog tested positive serologically but was negative by PCR, possibly reflecting prior exposure without active infection (45). Conversely, another case was PCR-positive but seronegative, which may be explained by low assay sensitivity, low antibody titers, or a prolonged sub-patent infection. Molecular testing targeting ITS1 loci and minicircle kDNA proved highly sensitive due to their elevated copy numbers, allowing detection even at low parasitemia levels. The use of ITS1 and hsp70 markers enabled confirmation of *L. infantum* in nearly all cases. The only exception was case 7, in which no blood sample was available. Altogether, combining serological and molecular methods improved diagnostic accuracy, highlighting the value of using complementary approaches to detect infection and better understand disease presentation in dogs.

The kDNA analysis provided relevant epidemiological insights, as it traced our sequences to the Old World, rather than the New World strains, which were introduced to the Americas approximately five centuries ago with European colonization (46). The New World strain has been confirmed in nearby countries such as Honduras (11, 47), Guatemala, and Panama. However, the sequences gathered in the present study all clustered with those from the Old World and shared haplotypes with them rather than with sequences from Brazil. Since the parasites could be traced back to their potential geographical origin, it would be relevant to study the sand fly hosts infected by the parasites in Costa Rica.

*Lutzomyia longipalpis* has been reported as the most abundant sandfly in Guanacaste, Costa Rica (48); however, other species may be implicated in the transmission of *L. infantum* in the region (49). Systematic sampling of sand flies and detection of *Leishmania* DNA will pinpoint the biological role of phlebotomines in the life cycle of *L. infantum* in Costa Rica. In this way, public health measures should be taken to decrease transmission in this and other geographical areas with confirmed vector distribution. In addition, gaining information regarding vector use will improve public health measures to prevent parasite transmission to humans.

The detection of positive dogs highlights the urgent need to establish control measures in Costa Rica. Although combination therapy with antimoniates and allopurinol is advised for treating CVL (50), the use of antimonials approved for humans is prohibited in veterinary medicine due to concerns about resistance development (51). This ban creates public health and ethical challenges, and leaves allopurinol as the only therapeutic option. Recommended by the World Health Organization for CVL (52), allopurinol is widely used for long-term parasite control. However, resistance has been reported, leading to relapses in naturally infected dogs (53). In countries such as Brazil, control relies on culling of seropositive dogs, though this has not reduced human or canine incidence (54). Instead, preventing sand fly bites has been recommended with spot-on or insecticide-impregnated collars (55). Vaccination against *L. infantum* has also been implemented as a preventive strategy (56), but high costs limit its broad application in endemic regions.

## 5 Conclusion

The findings of this study suggest that *L. infantum* parasites identified in Costa Rican dogs are more closely related to Old World strains, indicating a likely introduction from outside the Americas. This highlights the urgent need to improve surveillance, diagnostic capabilities, and treatment protocols for CVL in Costa Rica. Implementing routine screening in domestic dogs, vector surveillance, ensuring access to appropriate treatment and vaccine options, planning for management protocols for infected dogs, and establishing *L. infantum*-free certification for imported animals from endemic regions are essential steps toward controlling the spread of the parasite. These measures are crucial for reducing the risk of zoonotic transmission and protecting both animal and human health in the region. A comprehensive, One Health-based surveillance program that integrates data from a larger number of humans, dogs, wildlife reservoirs, and vector samples is crucial to substantiate our hypothesis and inform strategies for mapping parasite distribution and host–parasite dynamics.

## Data Availability

The datasets presented in this study can be found in online repositories. The names of the repository/repositories and accession number(s) can be found in the article.

## Supplementary material

SUPPLEMENTARY DATA SHEET 1 Demographic, clinical, laboratory and treatment information of all cases analyzed in the present study.

SUPPLEMENTARY DATA SHEET 2 Demographic and clinical Information of dogs living in the community of cases 2, 3, 4 and 5.

SUPPLEMENTARY DATA SHEET 3 Primers and PCR conditions used for the amplification of ITS-1, hsp70 and kDNA fragments of *Leishmania* spp.

SUPPLEMENTARY VIDEO 1 Histopathological analysis of spleen biopsy of Case 6 showing multiple intracellular *Leishmania* sp. amastigotes.

SUPPLEMENTARY IMAGE 1 Bayesian inference phylogenetic tree of a fragment of the heat shock protein 70 (hsp70) of *Leishmania* spp. *Leishmania* spp. are color-coded in the outer circle. The sequence derived from this study is denoted with a black diamond. Circle node size and color are proportional to the posterior probability values and values below 0.6 are not shown.
