# Supplementary material for: Emergence of autochthonous Leishmania infantum infection in dogs from Costa Rica confirmed by multimodal diagnostics: a case series
Source: Front Vet Sci. 2026 Jan 21;12:1704403. doi: 10.3389/fvets.2025.1704403 (PMC12870656; doi:10.3389/fvets.2025.1704403)
Supplement: SUPPLEMENTARY DATA SHEET 1 — Demographic, clinical, laboratory and treatment information of all cases analyzed in the present study. [file Data_Sheet_1.docx]

| **Case** | **Breed, Sex, Age** | **Location** | **Clinical Signs** | **Diagnostics** | **Hematology / Biochemistry** | **Treatment*** | **Outcome** |
| --- | --- | --- | --- | --- | --- | --- | --- |
| **1** | Mongrel, M, ≥4 y | Tamarindo, Santa Cruz, Guanacaste | Weight loss, cachexia, snout ulcer, hindleg alopecia | CBC, biochem, serology, PCR (EDTA blood, lymph node, skin, swabs) | Anaemia (HCT: 28.8%), Thrombocytopenia (155k), Azotemia (CREA: 3.9, BUN: 57) | Meglumine antimoniate 100 mg/kg SC 28d; Allopurinol 10 mg/kg PO BID 2mo | Lost after follow-up |
| **2** | Schnauzer, F, 4 y | Tamarindo, Santa Cruz, Guanacaste | None | Serology, PCR | Not specified | None (owner refused) | Died post-partum |
| **3** | Mongrel, F, 2 y | Tamarindo, Santa Cruz, Guanacaste | None | Serology, PCR | Not specified | None (owner refused) | Died (hit by car) |
| **4** | Mongrel, M, 8 y | Tamarindo, Santa Cruz, Guanacaste | None | Serology, PCR | Not specified | Allopurinol + Meglumine antimoniate | Unknown |
| **5** | Mongrel, M, 3 y | Tamarindo, Santa Cruz, Guanacaste | Chronic hindleg skin lesion | Serology, PCR | Not specified | Allopurinol only | Died (post-surgical complication) |
| **6** | Mongrel, F, 5 y | La Garita Nueva, Tamarindo | Exfoliative dermatitis, alopecia, crusts, onychogryphosis | CBC, serology, PCR (EDTA blood, skin) | Anaemia (HCT: 19.2%), Leukopenia (3.53k), Thrombocytopenia (77k) | Allopurinol 10 mg/kg PO BID 4mo | Ongoing treatment |
| **7** | Mongrel, F, 4 y | Born in Playa Grande, Santa Cruz, Guanacaste, moved to Santa Ana, San José | Ear and leg dermatitis | Cytology, histopathology, PCR (EDTA blood) | Not specified | Allopurinol 10 mg/kg PO BID | Lesions resolved |
| **8** | Boxer, F, 4 y | Tamarindo, Santa Cruz, Guanacaste (inspected in San José) | Splenomegaly, anaemia | CBC, splenectomy histopathology, PCR (EDTA blood) | Anaemia (HCT: 21.7%), Thrombocytopenia (113k) | Allopurinol + treatment for *Ehrlichia* and *Dirofilaria immitis* | Died post-splenectomy |

F: female, M: male, CBC: complete blood-count, HCT: hematocryt, CREA: creatinine, BUN: blood urea nitrogen, SC: subcutaneous, PO: oral, BIS: twice-daily. *Commercial name of meglumine antimoniate was Glucantime, Merial®, Germany for and allopurinol was Laboratorios Normon, S.A., Spain)
