# Supplementary material for: Emergence of autochthonous Leishmania infantum infection in dogs from Costa Rica confirmed by multimodal diagnostics: a case series
Source: Front Vet Sci. 2026 Jan 21;12:1704403. doi: 10.3389/fvets.2025.1704403 (PMC12870656; doi:10.3389/fvets.2025.1704403)
Supplement: SUPPLEMENTARY DATA SHEET 3 — Primers and PCR conditions used for the amplification of ITS-1, hsp70 and kDNA fragments of Leishmania spp. [file Data_Sheet_3.docx]

Supplementary file 3. Primers and PCR conditions used for the amplification of ITS1, hsp70 and kDNA fragments of *Leishmania* spp.

| Locus | Primers | Fragment size | PCR conditions | Reference |
| --- | --- | --- | --- | --- |
| ITS1 | ITS-219F (5´-AGCTGGATCATTTTCCGATG-3´)  ITS-219R (5´-ATCGCGACACGTTATGTGAG-3´) | 270 bp | Initial denaturation at 95 °C for 5 min, followed by 35 cycles of 95 °C for 10 s, 55 °C for 30s and 72 °C for 30 s, and a final amplification step at 72 °C for 7 min | [17] |
| Hsp70 | hsp70-F (5´-AGGTGAAGGCGACGAACG-3´)  hsp70-R (5´-CGCTTGTCCATCTTTGCGTC-3´) | 370 bp | Initial denaturation at 95 °C for 5 min, followed by 35 cycles of 95 °C for 1 min, 57 °C for 1 min and 72 °C for 1 min, and a final amplification step at 72 °C for 7 min | [19] |
| kDNA | MC1 (5´-GTTAGCCGATGGTGGTCTTG-3´)  MC2 (5´-CACCCATTTTTCCGATTTTG-3´) | 447 bp | Initial denaturation at 95 °C for 5 min, followed by 35 cycles of 95 °C for 1 min, 58 °C for 1 min and 72 °C for 1 min, and a final amplification step at 72 °C for 7 min | [18] |
