# Supplementary figures and images for: Emergence of autochthonous Leishmania infantum infection in dogs from Costa Rica confirmed by multimodal diagnostics: a case series
Source: Front Vet Sci. 2026 Jan 21;12:1704403. doi: 10.3389/fvets.2025.1704403 (PMC12870656; doi:10.3389/fvets.2025.1704403)

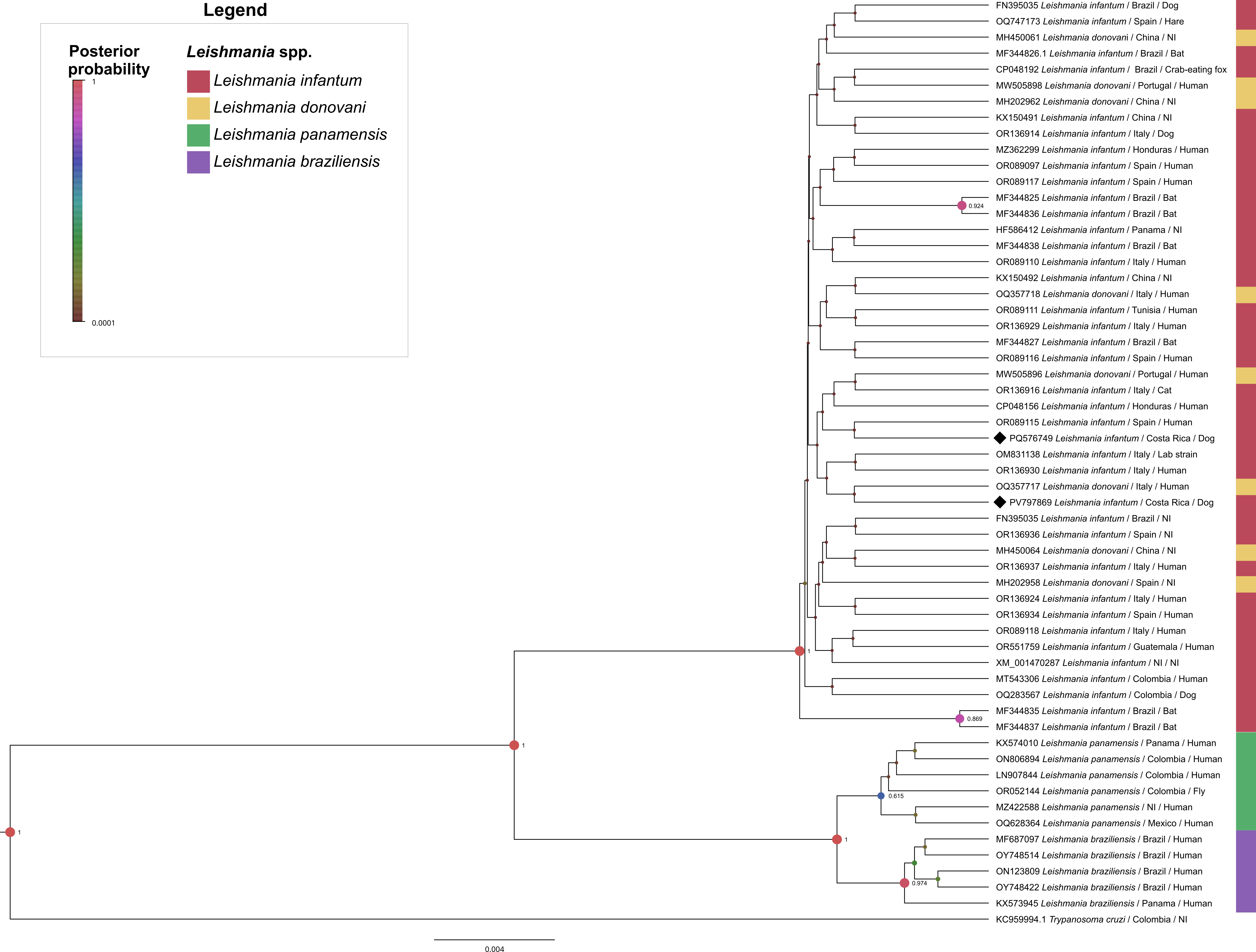

Supplement: SUPPLEMENTARY IMAGE 1 — Bayesian inference phylogenetic tree of a fragment of the heat shock protein 70 (hsp70) of Leishmania spp. Leishmania spp. are color-coded in the outer circle. The sequence derived from this study is denoted with a black diamond. Circle node size and color are proportional to the posterior probability values and values below 0.6 are not shown. [file Image_1.png]
